# Barriers to and Facilitators for Optimal Intervention for Apathy in Older Adults With Dementia

**DOI:** 10.1093/geroni/igab046.1083

**Published:** 2021-12-17

**Authors:** Aderonke Agboji, Shannon Freeman

**Affiliations:** 1 University of Northern British Columbia, Quesnel, British Columbia, Canada; 2 University of Northern British Columbia, Prince George, British Columbia, Canada

## Abstract

Apathy is a persistent symptom in brain disorders. It affects 84% of people with brain disorders. Those affected are more than two times likely to die early than those without. Yet it is often ignored and undertreated. An integrative review guided by Whittemore and Knafl's (2005) framework was carried out to identify the factors that inhibit or facilitate the diagnosis and management of apathy among older adults with dementia. The result of the findings revealed five barriers and three facilitators. Barriers included inconsistencies in the definition and diagnostic criteria, lack of awareness, overlap with other neuropsychiatric disorders, a paucity of evidence-based information, and lack of familiarity. Facilitators were standardized definition and assessment tools, good communication among the interdisciplinary team, and adequate training, education, and experience. In conclusion, efforts should be geared towards raising awareness and developing a practice guideline to aid healthcare professionals in detecting and managing apathy optimally.

